# Going the distance: validation of Acuros and AAA at an extended SSD of 400 cm

**DOI:** 10.1120/jacmp.v17i2.5913

**Published:** 2016-03-08

**Authors:** Narottam Lamichhane, Vivek N. Patel, Matthew T. Studenski

**Affiliations:** ^1^ Department of Radiation Oncology University of Miami Miami FL USA

**Keywords:** total body irradiation, extended SSD, AAA, Acuros

## Abstract

Accurate dose calculation and treatment delivery is essential for total body irradiation (TBI). In an effort to verify the accuracy of TBI dose calculation at our institution, we evaluated both the Varian Eclipse AAA and Acuros algorithms to predict dose distributions at an extended source‐to‐surface distance (SSD) of 400 cm. Measurements were compared to calculated values for a 6 MV beam in physical and virtual phantoms at 400 cm SSD using open beams for both 5×5 and $40\times 40\;{\text{cm}}^{2}$ field sizes. Inline and crossline profiles were acquired at equivalent depths of 5 cm, 10 cm, and 20 cm. Depth‐dose curves were acquired using EBT2 film and an ion chamber for both field sizes. Finally, a RANDO phantom was used to simulate an actual TBI treatment. At this extended SSD, care must be taken using the planning system as there is good relative agreement between measured and calculated profiles for both algorithms, but there are deviations in terms of the absolute dose. Acuros has better agreement than AAA in the penumbra region.

PACS number(s): 87.55.kd

## I. INTRODUCTION

The ideal delivery of total body irradiation (TBI) involves a uniform dose distribution to the whole body with a generally accepted uniformity within ±10% of the prescribed dose.^(1)^ This technique, along with chemotherapy, is most often utilized to treat myeloid and lymphoid disease by providing conditioning for bone marrow transplant.^(2)^ Radiation results in immunosuppression and also eradicates a modest number of radiosensitive tumor cells, clearing the host marrow to allow repopulation with donor marrow cells.^(3)^ Thereafter, hematopoietic stem cell transplantation may occur. It should be noted that although TBI offers a therapeutic opportunity for bone marrow transplantation, this benefit may be diminished by toxicity. Acute side effects include fatigue, mucositis, nausea, vomiting, loss of appetite, and diarrhea.^(4)^ In recent years, toxicity and quality of life measures have also been evaluated in patients who have survived for extended periods of time after TBI. The long‐term sequela of TBI may be associated with substantial chronic morbidities including pneumonitis, infertility, hypothyroidism, cataracts, and the possible risk of secondary malignancy.^(5)^

Various techniques to deliver uniform dose have been proposed in recent years such as step translation, dynamic field matching, volumetric arc therapy, and use of compensators.^3^, ^5^, ^6^, ^7^ Parallel‐opposed anterior/posterior and bilateral fields at extended source‐to‐surface distances (SSD) are commonly used as they offer a simple treatment solution. Dynamic techniques such as the sweeping beam method and the patient translation method were also developed in order to improve dose homogeneity and patient comfort although these techniques are more complex, typically are done at a shorter SSD, and might not be feasible in all clinics.^8^, ^9^, ^10^, ^11^

Regardless of the technique utilized, accurate dose calculation is essential to both treatment planning and the precise delivery of total body irradiation (TBI). Due to the aforementioned toxicity, it is imperative to ensure accurate dose delivery^3^, ^12^ to make sure that the prescription dose is delivered both to adequately immunosuppress the body and also ensure that the dose to critical structures is kept within tolerance to avoid injury — especially in the lungs.

The TBI dose calculation at an extended SSD is typically based on a point‐dose calculation approach.^(3)^ Although this method can be effective, unlike a treatment plan calculated in a treatment planning system (TPS), the point‐dose calculation technique fails to provide a full picture of the dose distribution in the patient, which can be critical in understanding the dose to sensitive structures prone to toxicity such as the lungs. Although the TPS seems to be the obvious solution to the problem, most radiation therapy occurs at SSDs of between 70 and 115 cm, not 400 cm; therefore, the commercial TPS is not designed or commissioned to calculate doses at an extreme, extended SSD.

The goal of this study is to assess the dose calculation accuracy of the Eclipse analytic anisotropic algorithm (AAA) and Acuros algorithms (Varian Medical Systems, Palo Alto, CA) at an extended SSD of 400 cm in both heterogeneous and homogeneous media. Other studies have explored this subject utilizing the AAA and ADAC Pinnacle (Philips Healthcare, Andover, MA) albeit at much shorter SSDs of less than 180 cm.^2^, ^13^ This study is unique in that two commissioned dose calculation algorithms are compared to measurements at an extended SSD of 400 cm.

## II. MATERIALS AND METHODS

### A. Dose calculation algorithms

All dose calculations were completed in Eclipse v. 11.0.47 using the analytic aniostropic algorithm (AAA) and the Acuros algorithm, both commissioned for clinical use. An energy of 6 MV was used, the calculation grid size was 2.5 mm, and all heterogeneity corrections were turned on. In Acuros, dose to medium was used. Plans were normalized to match the number of monitor units (MU) delivered at the linac during the various measurements. The source‐to‐surface distance (SSD) or the source‐to‐axis distance (SAD) in Eclipse was matched to the setup at the linac during the measurements.

### B. Measured to calculated dose comparison

The goal of this work was to assess the accuracy of the dose calculation algorithms at an extended SAD of 400 cm. This geometry posed problems as our clinical field size of $40\times 40\;{\text{cm}}^{2}$ projected a field size of $160\times 160\;{\text{cm}}^{2}$ at this distance. This not only made it difficult to acquire data due to the large field size, but it was difficult to find a phantom that was large enough to provide adequate scatter equilibrium. Therefore, in this work, we used a $5\times 5\;{\text{cm}}^{2}$ field size for most of the measurements, which projected a field size of $20\times 20\;{\text{cm}}^{2}$ at 400 cm SAD. This was much more manageable for acquiring relevant information and allowed for scatter equilibrium in the phantom. We did acquire data with the clinical field size for comparison although the goal here was not to commission a clinical system, which would require additional measurements.

#### B.1 Inline and crossline profiles

Measured profiles were obtained with an IBA Blue Phantom2 (IBA Dosimetry, Schwarzenbruck, Germany) water tank using two CC13 ion chambers (IBA Dosimetry); one field detector and one reference detector to reduce the effect of instantaneous machine output fluctuation. The water tank was set up with a 400 cm SSD to mimic the location of the patient in our TBI delivery. The field size was set to $5\times 5\;{\text{cm}}^{2}$ to project a field of $20\times 20\;{\text{cm}}^{2}$ at 400 cm SAD.

This geometry posed a practical problem as the beam entered through the side of the tank rather than the through the water surface. As the beam entered the tank through the plastic wall,

we corrected the depth of the profiles to account for the change in density (1.18 g / cm^3^). Inline and crossline profiles were obtained at equivalent depths of 5, 10, and 20 cm. The scanning speed was reduced to 0.3 cm / s to reduce noise. In Eclipse, the same beam geometry was created using a $40\times 40\times 40\;{\text{cm}}^{3}$ virtual water phantom and the dose was calculated using both algorithms. Profiles at the three respective depths were extracted. The calculated and measured profiles were plotted together to analyze any deviations at the extended SSD.

#### B.2 Percent depth‐dose curves

Due to geometric and physical limitations of the scanning water tank imposed by the wall, we measured the PDDs in two ways; first using Gafchromic EBT2 film (Ashland Inc., Covington, KY) and second using a PTW parallel plate ion chamber (PTW‐Freiburg GmbH, Freiburg, Germany) in a $30\times 30\times 30\;{\text{cm}}^{3}$ Solid Water slab phantom. The PDDs for both 5×5 and $40\times 40\;{\text{cm}}^{2}$ field size were acquired. For the film measurement, the phantom was setup at a SSD of 400 cm SSD and a strip of Gafchromic EBT2 film was sandwiched between two slabs parallel to the beam central axis. 5000 MUs were delivered for each field size and we scanned the irradiated film using an Epson v700 flatbed scanner (Epson America Inc., Long Beach, CA). We obtained a profile using the ImageJ software package (http://www.imagej.nih.gov) and the values from the red channel, only.

For the ion chamber measurements, the chamber was placed in a milled piece of solid water and we acquired readings at depths of 0, 0.5, 1, 1.5, 2, 3, 5, 7, 10, 15, 20, and 26 cm, keeping the phantom SSD at 400 cm. We extracted the PDD from Eclipse by obtaining a profile across a Solid Water phantom for both AAA and Acuros. We normalized all PDDs so that the maximum dose was 100%.

#### B.3 Absolute point dose

To assess the accuracy of AAA and Acuros at calculating doses at extended SSDs including heterogeneous media, we utilized several different phantoms. First, two homogeneous Solid Water phantoms, 19 and 31 cm thick, were used to simulate different patient separations with the central slab holding a Farmer‐type Exradin A12 ion chamber (Standard Imaging, Middleton, WI). Second, a heterogeneous phantom was made from a 17 cm thick Styrofoam block sandwiched between two solid water slabs of 2 cm (anterior) and 3 cm (posterior) with a slot cut for the A12 ion chamber in the Styrofoam phantom.

We scanned the phantoms on our Siemens Somatom Sensation CT simulator (Siemens Medical Solutions USA, Malvern, PA) using the clinical pelvis protocol (2 mm slice thickness, 120 kVp, 425 mAs) and sent to Eclipse. The phantoms were scanned twice, once with the ion chamber in place and once without the chamber to assess the effects of the presence of the chamber. We setup the phantoms with the chamber at 400 cm SAD and irradiated them with $5\times 5\;{\text{cm}}^{2}$ and $40\times 40\;{\text{cm}}^{2}$ field sizes, 1000 MU each.

We mimicked this beam delivery geometry in Eclipse and the dose to the ion chamber was calculated by contouring the active volume and finding the mean dose to this volume calculated with AAA and Acuros. We recorded the dose calculated on both the scans with and without the chamber present to assess the accuracy of each algorithm accounting for the perturbation of the ion chamber. These doses were compared to the measured dose delivered at the linac.

#### B.4 RANDO phantom

We setup the RANDO phantom (The Phantom Laboratory, Salem, NY) with a 400 cm SAD to the umbilicus midplane and a $40\times 40\;{\text{cm}}^{2}$ field size to mimic our clinical TBI delivery. EBT2 films were placed between two different slices in the RANDO phantom; the level of the superior lung (slice 12) and the level of the lower lung (slice 18). We delivered 1741 MUs per field equating to about 215 cGy at the midplane of the phantom at the umbilicus level from AP and PA beams.

To convert the film optical density to dose, we irradiated six films to doses of 0, 50, 175, 200, 225, and 250 cGy using the Solid Water phantom at a SAD of 400 cm to account for any energy dependence in the film. All films were scanned using the Epson v700 and the red color channel, only. The films were converted to dose in the Dose Lab Pro software (Mobius Medical Systems, Houston, TX).

We scanned the RANDO phantom on the Siemens CT scanner and the AP/PA beam geometry was mimicked in Eclipse. The dose planes from slice 12 and 18 were extracted from Eclipse to be compared to the film measurements. Using the Dose Lab Pro software, relative gamma analysis was performed on the two different planes for both AAA and Acuros. The gamma analysis was completed using both a 3% dose difference/3 mm distance to agreement and 5% dose difference/3 mm distance to agreement.

## III. RESULTS

### A. Inline and crossline profiles

**Figure 1:**
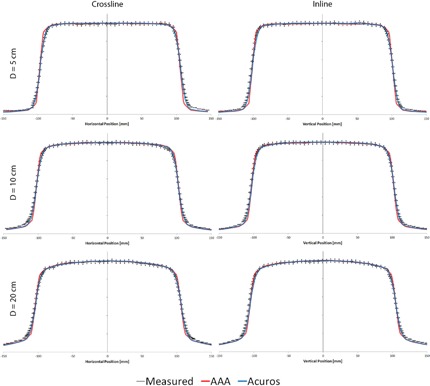
Measured (black with error bars) inline and crossline profiles at three depths compared to AAA (red) and Acuros (blue) calculated profiles at the same depths. The error bars represent 2% and 2 mm errors.

Figure 1 shows the comparison between the measured and calculated inline and crossline profiles at the three depths. There is good agreement between both algorithms and the measured data (<2% and 2 mm), except in the out‐of‐field region and in the transition regions of the penumbra. The out‐of‐field region might not affect the dose distribution depending on the TBI delivery method. For example, in our clinic patients are treated with $40\times 40\;{\text{cm}}^{2}$ fields at an extended SSD so the effect of the out‐of‐field region is minimized.

**Figure 2:**
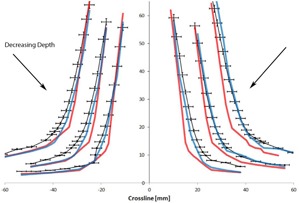
Comparison of crossline measured (black) with AAA (red) and Acuros (blue) profiles at 5, 10, and 20 cm depths zoomed in on the lower transition region of the penumbra. The error bars represent 2% and 2 mm errors. The absolute positions of the profiles have been shifted for display purposes.

**Figure 3:**
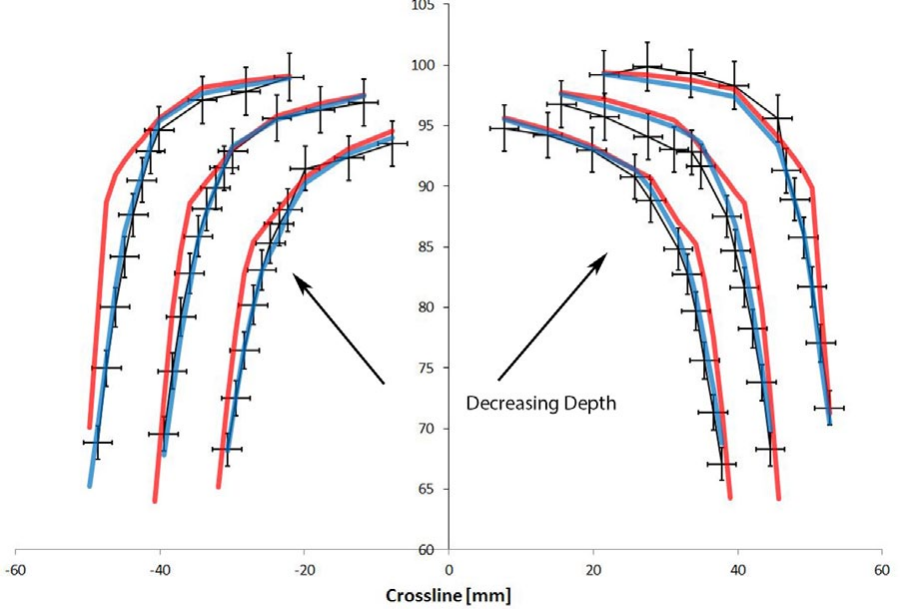
Comparison of crossline measured (black) with AAA (red) and Acuros (blue) profiles at 5, 10, and 20 cm depths zoomed in on the upper transition region of the penumbra. The error bars represent 2% and 2 mm errors. The absolute positions of the profiles have been shifted for display purposes.

**Figure 4:**
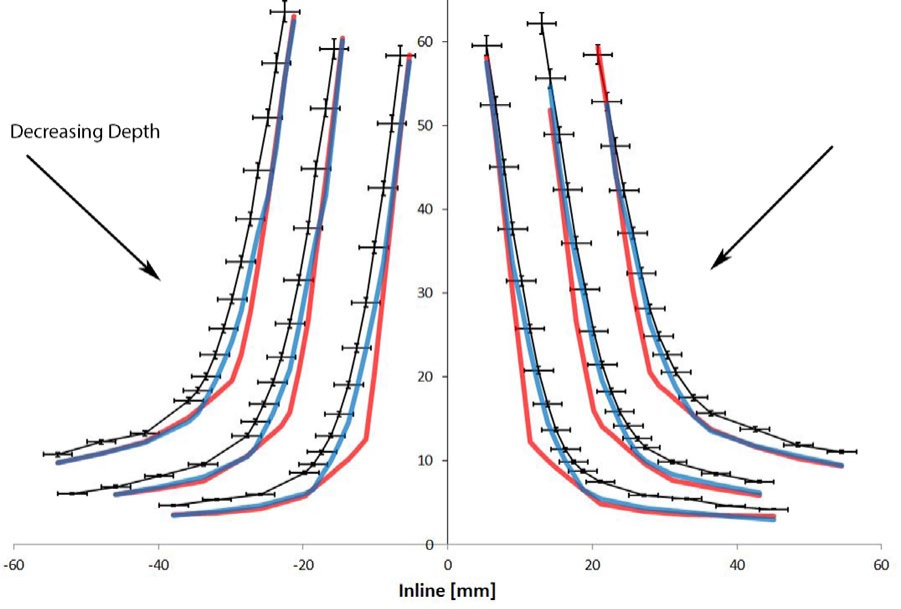
Comparison of inline measured (black) with AAA (red) and Acuros (blue) profiles at 5, 10, and 20 cm depths zoomed in on the lower transition region of the penumbra. The error bars represent 2% and 2 mm errors. The absolute positions of the profiles have been shifted for display purposes.

**Figure 5:**
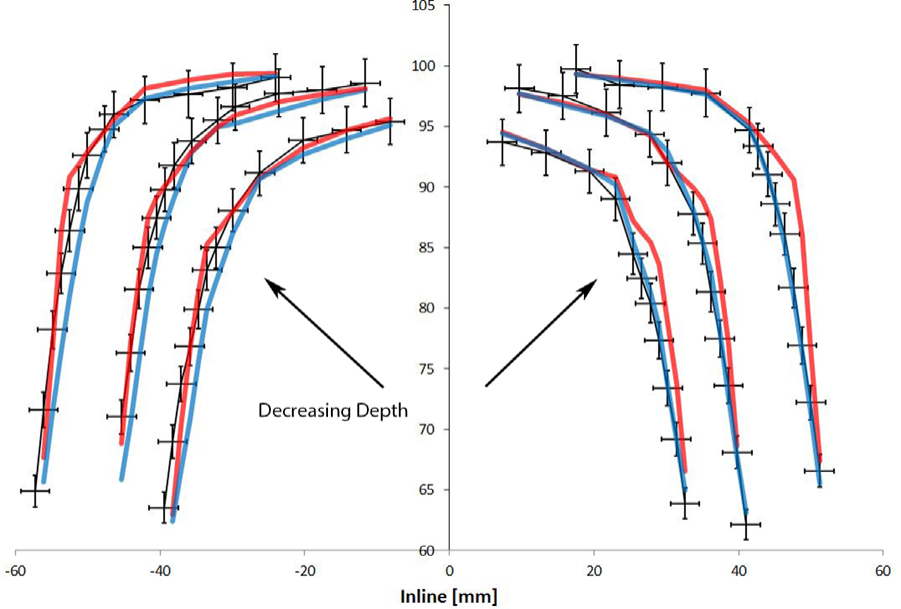
Comparison of inline measured (black) with AAA (red) and Acuros (blue) profiles at 5, 10, and 20 cm depths zoomed in on the upper transition region of the penumbra. The error bars represent 2% and 2 mm errors. The absolute positions of the profiles have been shifted for display purposes.

The areas of interest in the profiles are the transition regions where there are discrepancies larger than 2% or 2 mm between the calculated and measured profiles. Figures 2–5 focus on these regions to better visualize the differences between the measured and calculated data. At all depths, Acuros matches with the measured profile better than AAA.

### B. Percent depth‐dose curves

**Figure 6:**
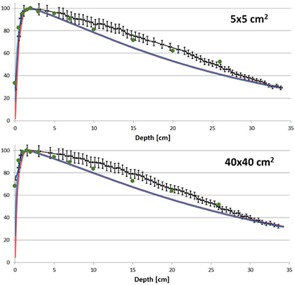
Comparison of the film measurements (black) and ion chamber readings (green dots) with PDDs calculated in AAA (red) and Acuros (blue) for two field sizes. The error bars represent 5% and 2 mm errors.

Figure 6 shows the comparison of the measured and calculated PDD curves. The PDDs from Acuros and AAA are almost identical but both underestimate the dose by more than 5% for clinically relevant doses between 8 and 30 cm deep. The film and ion chamber measurements agreed to within 5% for all depths. The deviation in the measured and calculated PDDs means that using either algorithm for absolute dose assessment or to calculate MUs for treatment is not recommended.

### C. Absolute point dose

Table 1 contains the results of the absolute dose measurements with the Solid Water phantoms and heterogeneous phantom. In the Solid Water phantom, both algorithms overestimated the dose by up to 4.9%, while in the heterogeneous phantom the dose was underestimated by both algorithms by up to 27.6%. This result further supports the recommendation that these algorithms should not be used for absolute dose calculations or MU determination at extended SSDs.

**Table 1:** Measured and calculated point doses in the heterogeneous Solid Water and Styrofoam phantom

		$5\times 5\;{\text{cm}}^{2}$			$40\times 40\;{\text{cm}}^{2}$		
		*Measured (cGy)*	*Calculated (cGy)*	*% Diff*.	*Measured (cGy)*	*Calculated (cGy)*	*% Diff*.
AAA	19 cm solid water w/chamber	50.7	53.1	4.7	58.5	60.2	2.9
	19 cm solid water no chamber	50.7	52.4	3.4	58.5	59.5	1.7
	31 cm solid water w/chamber	41.98	42.9	2.2	49.5	50.2	1.4
	31 cm solid water no chamber	41.98	43.0	2.4	49.5	50.9	2.8
	Styrofoam w/chamber	56.8	54.1	−4.8	64.7	63.3	−2.2
	Styrofoam no chamber	56.8	49.8	−12.3	64.7	61.9	−4.3
Acuros	19 cm solid water w/chamber	50.7	53.1	4.7	58.5	59.6	1.9
	19 cm solid water no chamber	50.7	53.2	4.9	58.5	59.7	2.1
	31 cm solid water w/chamber	41.98	43.8	4.3	49.5	50.7	2.4
	31 cm solid water no chamber	41.98	43.8	4.3	49.5	50.8	2.6
	Styrofoam w/chamber	56.8	55.1	−3.0	64.7	61.7	−4.6
	Styrofoam no chamber	56.8	41.1	−27.6	64.7	53.9	−16.7

Acuros demonstrated more sensitivity to perturbations in material density than AAA. When the chamber was included in the CT image in Acuros, the calculated dose changed by up to 1.3% in the Solid Water phantom. In the heterogeneous phantom, the change in the dose calculated with and without the chamber present was up to 25%. In comparison, in AAA it was only up to 16%. This extra sensitivity in Acuros is due in part to the ability to account for physical processes in dose deposition rather than simply modeling them like AAA.^(14)^

### D. RANDO phantom

**Figure 7:**
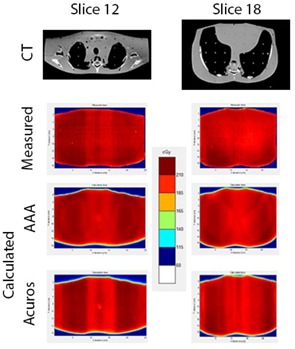
CT images of slices 12 and 18, along with the measured film dose and AAA and Acuros calculated dose distributions.

**Figure 8:**
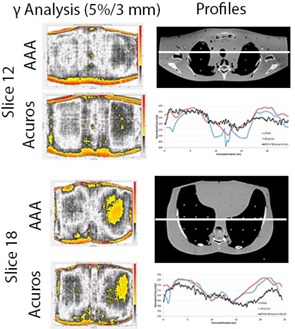
Results of the gamma analysis (5% dose difference, 3 mm distance to agreement) and profiles for RANDO slices 12 and 18. Notice that the failing points (color) are at the edge and outside of the phantom.

Figure 7 shows the CT images and the calculated and measured dose distributions for slices 12 and 18 in the RANDO phantom. Notice that the film is not as sensitive as the planning system for picking up details in the distribution but the increased dose in the lung region is still noticeable. Figure 8 shows the results of the gamma analysis (5% dose difference, 3 mm distance to agreement) and profiles for both slices. Due to the absolute dose difference between the calculated and measured dose distribution, relative gamma analysis was performed. Table 2 contains all the gamma passing rates, demonstrating the results from AAA and Acuros are comparable across the board.

The majority of the failing points are found around the periphery of the phantom. This is attributed to the fact that the edge of the film extended beyond the phantom and, therefore, some dose was delivered to the film, whereas in Eclipse, no dose is deposited outside of the phantom. The points inside the phantom showed much better agreement. The profiles in Fig. 8 show that the planning system is much more sensitive to tissue heterogeneities than the film measurement, but the effect of the lung heterogeneity can be observed in all profiles.

**Table 2:** Results of the gamma analysis

		*3% DD, 3 mm DTA*	*5% DD, 5 mm DTA*
Slice 12	AAA	74.4%	87.4%
	Acuros	64.2%	85.9%
Slice 18	AAA	60.3%	81.5%
	Acuros	61.9%	86.5%

DD=dose difference, DTA=distance to agreement.

## IV. DISCUSSION

In this study, we evaluated AAA and Acuros at an extended SSD of 400 cm during TBI, which has not been previously investigated. TBI has an important role in conditioning patients for bone marrow transplant in that it both eradicates malignant cells and prevents rejection of donor hematopoietic cells via immunosuppression; however, it does have side effects that may lead to chronic morbidity. To assuage the risk of toxicity, treatment accuracy is of utmost significance, and the use of the treatment planning system can provide three‐dimensional dose distributions to assist with the clinical decision‐making.

Several studies have looked at the accuracy of various treatment planning systems and techniques, although none of them considered distances greater than 200 cm. Lavallee et al.^(15)^ studied the dose distribution using the Pinnacle^3^ planning system at 100 cm SSD for TBI using a moving couch, and compared measured data to the 3D pencil beam and the superposition convolution algorithm. Hussain et al.^(13)^ validated the Eclipse AAA algorithm at an extended SSD of 179.5 cm and compared it to commissioned and gold beam models. To our knowledge, the present study is the first to consider both the AAA and Acuros algorithm at an extended SSD greater than 180 cm.

The major finding of this study is that the Eclipse planning system tends to underestimate the dose delivered at extended SSDs. This can be seen in the PDDs and the heterogeneous point dose measurements. The relative dose distribution compares well with the measurements, as seen in the profiles and gamma analysis with the RANDO phantom. The results in this study show that AAA and Acuros are comparable in calculating relative isodose distributions at extended SSDs, with a slight edge given to Acuros in the penumbra region.

With this result in mind, the planning system should not be used to determine the MUs needed for a clinical TBI delivery and additional commissioning measurements are required to clinically implement a TBI program. The relative dose distributions can be used to aid in planning decisions, such as lung blocking.

One area not covered in this paper was the use of compensators, blocks, or scatter screens. We did not analyze these devices as the inclusion of high‐Z materials adds uncertainty to the calculated dose and modeling devices that are outside of the patient can be difficult. As the focus here is purely the accuracy of AAA and Acuros at an extended SSD, we felt these were beyond the scope of the paper and will be considered in ongoing research.

The validation of the dose calculation algorithms at extended SSDs of 400 cm opens the doors to more exotic TBI deliveries in the future, although additional commissioning is required to validate that the out‐of‐field differences do not compromise the delivered dose. For example, the MLC could be used to block the lungs to eliminate the need to cut blocks. This also opens the possibility of using a field‐in‐field technique to deliver a more homogenous dose. The greatest advantage of using the planning system is the ability to visualize a three‐dimensional dose distribution in the patient.

## V. CONCLUSIONS

The AAA and Acuros dose calculation algorithms were assessed for accuracy in calculating isodose distributions for TBI at an extended SSD. The acquired data were measured using various homogenous and heterogeneous phantoms. Results have shown that the relative dose distribution achieved with both AAA and Acuros agree well with measurements but, in absolute dose, there are deviations that can exceed 10%. Therefore, it is not recommended to use the Eclipse planning system to calculate MUs for TBI treatments at extended SSDs. The relative dose distributions can be used to assist with treatment planning decisions such as lung blocking. Acuros shows slightly better agreement in the penumbra region than AAA. These discoveries may subsequently lead to the creation of more novel dose calculation and treatment delivery techniques in the future.

## COPYRIGHT

This work is licensed under a Creative Commons Attribution 4.0 International License.
